# Diagnostic and therapeutic role of non-coding RNAs regulating programmed cell death in melanoma

**DOI:** 10.3389/fonc.2024.1476684

**Published:** 2024-12-24

**Authors:** Zixu Wang, Cong Xie, Xiao Chen

**Affiliations:** ^1^ Office for Doctoral Studies, Charité-Universitätsmedizin Berlin, Berlin, Germany; ^2^ Office for Postgraduate Student Studies, Kunming Medical University, Kunming, China

**Keywords:** programmed cell death, non-coding RNAs, melanoma diagnostic, therapeutic, melanoma

## Abstract

lncRNAs (long non-coding RNAs) are heterogeneous RNA molecules that modulate various cellular processes, such as proliferation, differentiation, migration, invasion, and apoptosis, via different mechanisms. An increasing amount of research indicates that abnormal expression of lncRNA influences the development of drug resistance as well as the genesis and advancement of cancer, including melanoma. Furthermore, they are attractive biomarkers for non-invasive cancer diagnostics due to their strongly modulated expression and improved tissue and disease specificity. This review offers a succinct overview of the present understanding concerning the potential diagnostic biomarker potential of lncRNAs in melanoma. Cell death occurs frequently during growth and throughout life and is an active, organized, and genetically determined process. It is essential for the regulation of homeostasis. Controlled cell death and non-programmed cell death are both forms of cell death. The most prevalent forms of regulatory cell death are pyroptosis, ferroptosis, autophagy, necroptosis, necrosis, and apoptosis. Ferroptosis, pyroptosis, and autophagy are less common forms of cell death compared to necrosis, apoptosis, and necroptosis. ncRNAs are regulatory RNA molecules that are not involved in encoding proteins. They primarily consist of circular RNAs (circ RNAs), lncRNAs, and microRNAs (miRNAs). Moreover, non-coding RNAs have the ability to modulate tumor cell autophagy, pyroptosis, and ferroptosis at the transcriptional or post-transcriptional stage, as well as function as oncogenes and tumor suppressor genes, which can have considerable effects on the incidence and growth of tumors. This review concentrated on the recent advancements in the research of the diagnostic and therapeutic functions of ncRNAs in the regulation of programmed cell death in melanoma.

## Introduction

Melanoma is the deadliest and utmost severe kind of skin malignancy. It has a low prognosis and comes from melanocytes (1, 2). Melanoma can be classified into four subgroups based on molecular features and clinical aspects: mucosal melanoma, acral melanoma, cutaneous melanoma deprived of chronic sun impairment, and cutaneous melanoma with chronic sun impairment (3, 4). The most common kinds of melanoma in white individuals are non-acral cutaneous melanomas while acral as well as mucosal melanomas are far less common, occurring in only 1% and 5% of cases, respectively (5, 6). It is well-established that acral and mucosal melanomas are more common in Asian populations compared to other melanoma subtypes, but the exact percentage can vary depending on the study, as one study reported about 70% (7).

**Figure 1:**
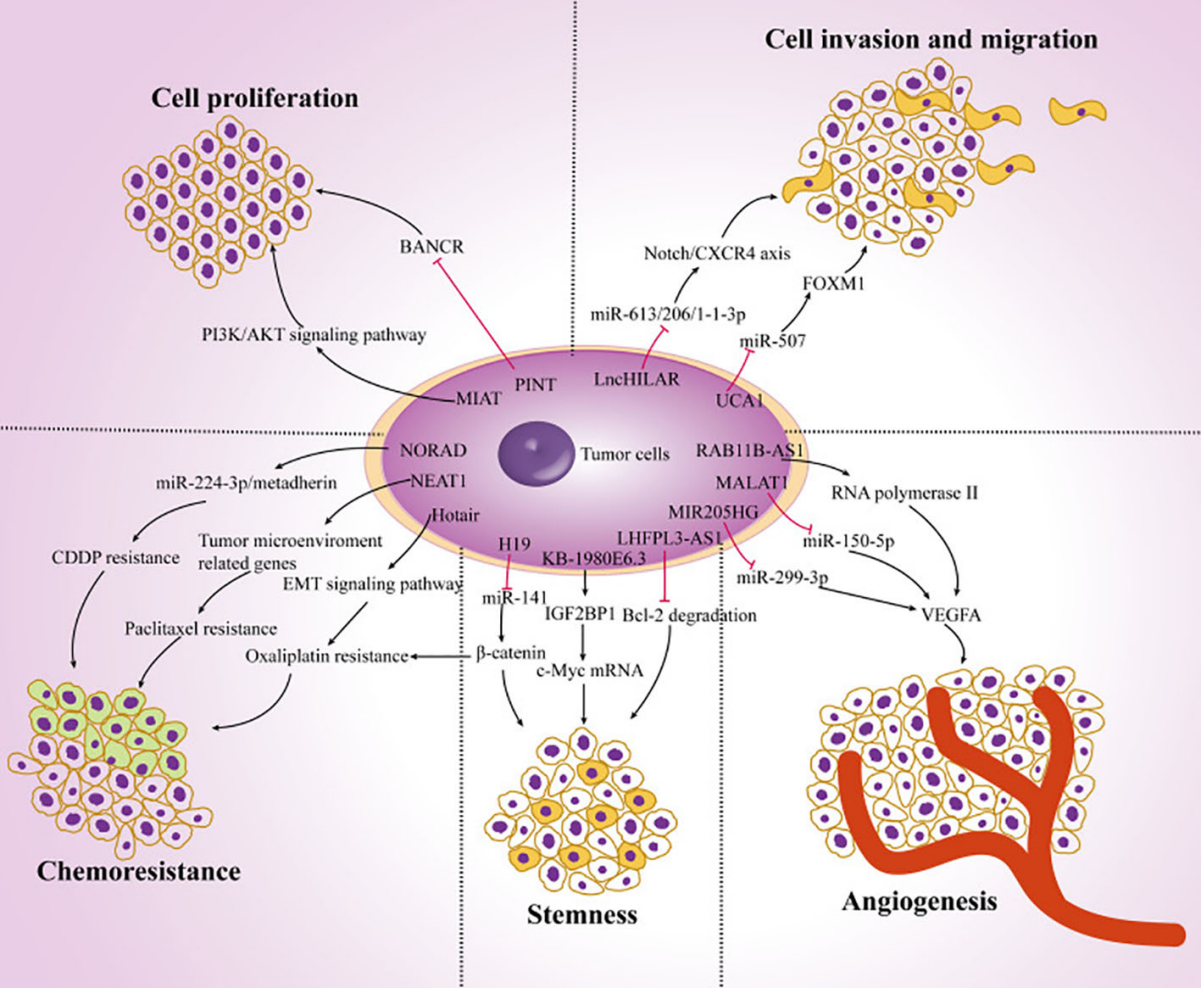
The function of lncRNAs in the development of cancer. Ultimately, the dysregulation of lncRNAs in melanoma cells regulates tumor progression by targeting multiple genes and affecting tumor cell proliferation, chemoresistance, stemness, angiogenesis, invasion, and migration. Reproduced with permission from (19).

The prevalence of melanoma is rising at a rate of 5% annually, according to recent statistics (8). Even though it only makes up 10% of skin cancers, up to 80% of them are fatal (9). The primary causes of the high death rate are the enhanced degree of malignancy, rapid progression, and the unsatisfactory nature of current therapies for melanoma. Patients may experience both local lymph nodes and distant organ metastases in the early stages. Clinically, melanoma can be identified and treated in a variety of ways. The most effective treatment methods at present are lymph node dissection, larger resection, and prompt detection (10). The outlook is low with a 5-year survival rate (< 20%) because an enormous proportion of persons have metastasized at the interval of identification (8). However, some patients have benefited significantly in the past years from the medical use of targeted and immunotherapy, particularly from targeted treatments like BRAF (B-Raf proto-oncogene, serine/threonine kinase; vemurafenib) as well as mitogen-activated protein kinase kinase (MEK) (trametinib) inhibiting agents (11–13). On the other hand, because of the subtype bias, over 50% of Asian patients cannot advantage from targeted treatment for c-Kit and BRAF. Furthermore, overall c-Kit and BRAF mutation frequencies are approximately 10.8% and 25.5%, respectively, because of this cohort’s poor tumor gene mutation burden (14, 15). Furthermore, clinical research has revealed that immunotherapy employing anti-programmed cell death 1 (PD1) or anti-cytotoxic T lymphocyte-associated protein 4 (CTLA4) antibodies offers significant medical advantages to several melanoma-diseased persons, while medication confrontation persists (16–18). Therefore, finding novel anti-tumor targets and melanoma-specific markers is significant in the domain of melanoma investigation. Numerous reports have demonstrated that lncRNAs are significant in the growth and progression of cancer. Tumor cell proliferation, metastasis, EMT, stemness, angiogenesis, chemotherapy resistance, and tumor microenvironment modulation are among the biological processes in which they are implicated (**Figure 1**).

ncRNAs represent a set of RNA that do not code for proteins; they make up 98% of the human genome and are primarily classified as miRNAs, lncRNAs, as well as circRNAs (20). Although ncRNAs are not tangled in encoding proteins, they are essential for cell growth and metabolism and have the ability to modulate physiological functions like protein translation, RNA transcription, and DNA replication. Their role as proto-oncogenes or tumor suppressor genes is crucial to modulate the occurrence and propagation of malignancies (21–24). Investigating the function of various ncRNAs in controlling the growth, metastasis, recurrence, angiogenesis, and migration of cancers has been the subject of more research in recent years, particularly in the context of melanoma (25, 26). In this report, current research on the function of circRNAs, lncRNAs, and miRNAs in melanoma is extensively discussed. Furthermore, a summary of the existing understanding of the connection between ncRNAs and melanoma in addition to discussing the significance and potential of ncRNAs in the identification as well as therapy of melanoma are discussed.

## Biological attributes and role of ncRNAs

**Figure 2:**
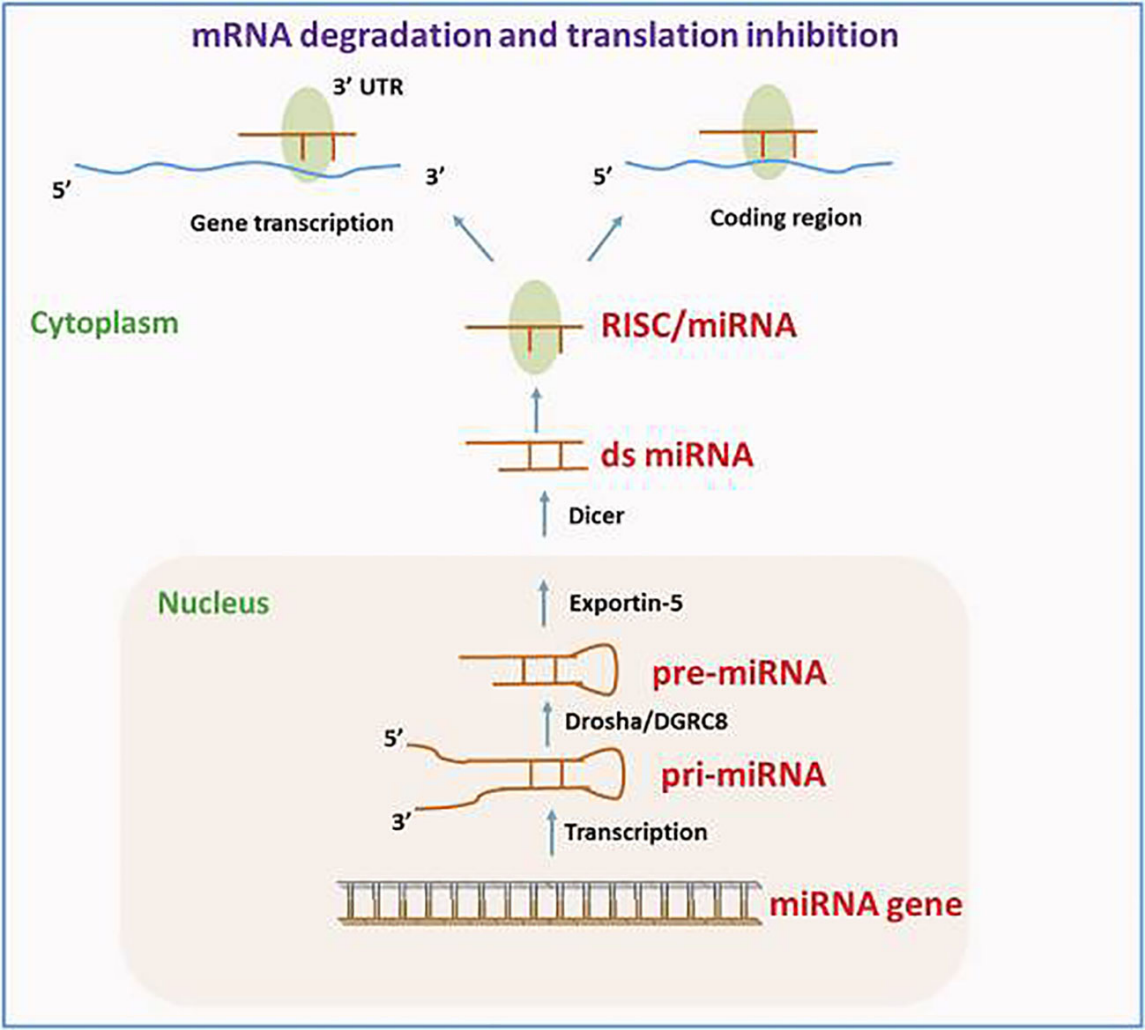
The route of miRNA biogenesis and miRNA in gene modulation. Reproduced with permission from (34).

Various reports have demonstrated that approximately 1.5% of the nucleic acid sequences in the human genome are associated with protein-coding (27–29). The remaining sequences are ncRNA, given their lack of involvement in encoding proteins. This discovery is made possible by the prompt establishment of next-generation sequencing technology and the growth of human genomics (30). The ncRNAs consist of competitive endogenous RNA (ceRNA), circRNA, lncRNA, miRNA, transfer RNA (tRNA), and ribosomal RNA (rRNA). Moreover, circRNA, lncRNA, and miRNA are among several ncRNAs that have been extensively demonstrated to have significant functions in the emergence as well as progression of tumors (31–33). The miRNAs, measuring approximately 18 –24 nt in length, represent a category of endogenous non-coding small RNAs. A large portion of miRNAs undergo transcription from introns or miRNA DNA. The miRNA sequence undergoes transcription into the primary transcript termed primary miRNA (pri-miRNA), following that RNA polymerase II caps and polyadenylates. Drosha then cuts the pri-miRNA in the nucleus for producing a precursor miRNA (pre-miRNA). Following that, the pre-miRNA is bound through ran-guanosine triphosphate (GTP) and exportin 5, facilitating its transfer from the nucleus into the cytoplasm. Pre-miRNA is broken down by Dicer into a developed, double-stranded, about 22-nt miRNA/miRNA duplex in the cytoplasm. Therefore, the RNA-induced silencing complex (RISC) attaches to a double-stranded miRNA complex. Following the complementary strand’s removal as well as degradation from the RISC, functional miRNA is generated. Its main mechanism of action is to form the miRISC complex by combining with RISC, and then it selectively binds to the mRNA that codes for the protein. This process either degrades the target mRNA or prevents it from being translated, controlling post-transcriptional gene expression (**Figure 2**) (35–37).

**Figure 3:**
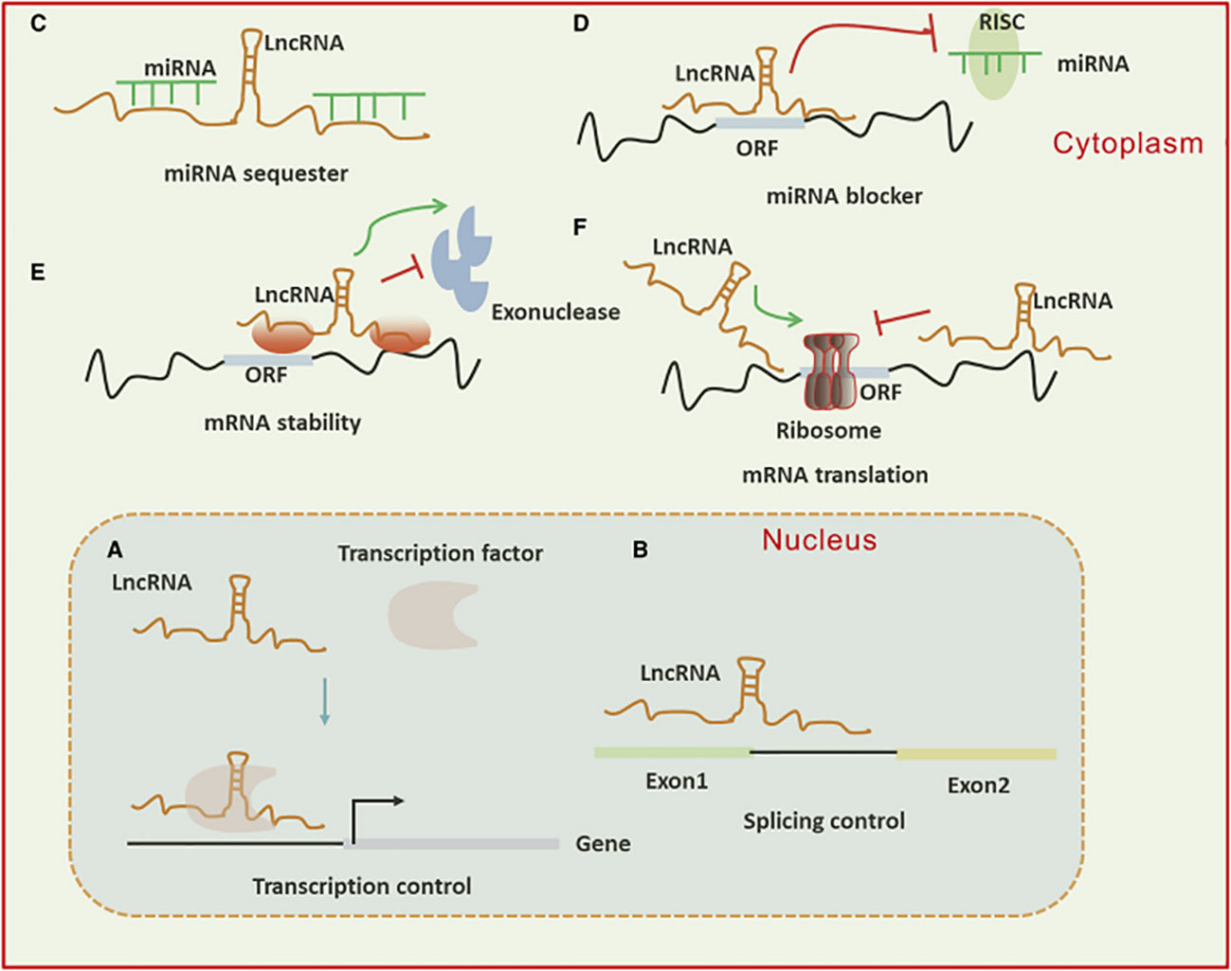
**(A)** Long non-coding RNAs (lncRNAs) can activate gene transcription by facilitating the recruitment of transcription factors to enhancers or promoters and suppress transcription by sequestering transcription factors away from these regulatory regions. **(B)** They influence mRNA function through base pairing, which can modify splicing patterns. **(C, D)** lncRNAs act as “sponges” by binding to complementary microRNAs (miRNAs), thereby inhibiting their activity. In the cytoplasm, lncRNAs regulate mRNA expression by competing with miRNAs for binding sites or blocking their function. **(E, F)** Additionally, lncRNAs modulate mRNA expression by affecting mRNA stability or translation through base pairing interactions in the cytoplasm.

A subset of ncRNAs featuring transcripts longer than 200 nt are known as lncRNAs. They can function as a “molecular sponge,” by competing with miRNAs, DNAs, or transcription factors to either up or down-regulate the expression of target proteins. Transcriptional output can be directly impacted by lncRNAs through their ability to either attract or inhibit the binding of transcription factors and transcriptional elements. Moreover, lncRNAs have a role in the nucleus’ post-transcriptional control. For instance, lncRNAs can control certain splicing patterns by interacting with the splicing equipment or with nascent RNAs. LncRNAs can affect translational output in the cytoplasm in a variety of manners. Firstly, they can influence the internal ribosomal entry sites or modulate polysome loading to an mRNA molecule to control the translational rate. Furthermore, they can govern the gene activity by either promoting or inhibiting mRNA degradation (**Figure 3**) (38–40). The circRNAs represent a new group of endogenous non-coding RNAs that lack a poly(A) tail at the 3′ end and a 5′ end cap and instead have a unique covalent closed loop structure. Various forms of circRNAs can be formed from introns or exons, including intronic, exonic, and exon-intron circRNAs. Pre-mRNA splicing produces the exonic circRNA. When the 3′ splice donor and 5′ splice acceptor are combined, the exonic circRNA is produced. The resultant circular transcript is known as exon-intron circRNA when the introns between the exons remain reserved. Intronic lariats, which are resistant to being broken down by debranching enzymes, are capable of producing intronic circRNAs. Their role as a miRNA sponge allows them to control the ceRNA process, protein translation, alternative splicing, and gene transcription (41–43).

## Function of lncRNAs in tumor cell mortality for tumorigenesis, growth, and treatment

### lncRNAs and autophagy

lncRNAs primarily play a role in autophagy and impact a variety of cancers, including pancreatic cancer, prolactinoma, osteosarcoma, OSCC, NPC, thyroid papillary carcinoma, PCa, ovarian malignancy, bladder cancer, NSCLC, HCC, hepatoma, chronic myelogenous leukemia (CML), glioma, glioblastoma, breast cancer (BC), gastric cancer, acute myelogenous leukemia (AML), CRC, and uveal melanoma. For instance, uveal melanoma cells express the lncRNA ZNF706 neighboring transcript 1 (ZNNT1) at a reduced extent. ZNNT1 induces autophagy by enhancing the expression of ATG12, which displays a tumor-inhibitory impact (44). One prominently conserved self-degradation mechanism in eukaryotic cells which is significant in controlling cell division, maturation, aging, and death is autophagy. Autophagy sometimes contributes to cell death, a process known as autophagic cell death. Lysosomes are used by autophagic cells to break down macromolecules and impaired organelles to preserve homeostasis. The cells initially produce a mono or bilayer membrane, which matures into an autophagosome that resembles a vesicle. This autophagosome combines with a lysosome to produce an autolysosome, which causes the material inside to be broken down by lysosomal hydrolase and the products to be recycled (45, 46).

Reduced expression of the lncRNA urothelial cancer-associated 1 (UCA1) prevents the growth and autophagy of CRC cells (47). EIF3J divergent transcript (EIF3J-DT) is significantly upregulated in gastric malignancy cells following exposure to chemotherapeutic medications. EIF3J-DT selectively modulates the levels of ATG14, resulting in the stimulation of autophagy, and thereby inducing medication resilience in gastric malignancy cells (48). LN18 and U138 cell lines contain the lncRNA growth arrest-specific 5 (GAS5), which improves cisplatin susceptibility by triggering mTOR signaling, thereby preventing autophagy (49). Glioma cells have increased levels of tumor protein translation regulator 1 antisense RNA 1 (TPT1-AS1), and autophagy is mediated by the miR-770-5p/TPT1-AS1/stathmin 1 (STMN1) alliance in glioma cells. This corresponds to the directed therapy of glioma (50). Tamoxifen-resistant MCF7 cells’ autophagy is prevented by downregulating H19 imprinted maternally expressed transcript (H19) activity. H19 causes autophagy via S-adenosyl-L-homocysteine hydrolase (SAHH)/DNA methyltransferase 3 beta (DNMT3B) alliance, enabling clarification of the biochemical trail of tamoxifen confrontation in BC (51). Decreased RNA in malignancy prevents glioblastoma-derived cell lines from invasively entering the cell and triggers adenosine monophosphate-triggered protein kinase (AMPK) through the downregulation of glucose transporter 1 (GLUT1) levels. This, in turn, suppresses the expression of mTOR and stimulates autophagy (52). Glioma cell movement and development are enhanced by H19 overexpression, and H19 stimulates glioma cell autophagy and proliferation via Unc-51/mTOR- like autophagy-triggering kinase 1 (ULK1) trail (53). The increased expression of HAGLROS in HCC could be associated with the mTOR/AKT/PI3K signaling trail and the ATG12/miC-5095 alliance, which are implicated in autophagy and apoptosis (54). The activities of metastasis-associated lung adenocarcinoma transcript 1 (MALAT1) are downregulated in HCC cells, which influences the expression of miR-146a and facilitates apoptosis and autophagy (55). The increased expression of HOX transcript antisense RNA (HOTAIR) improves the development of cervical malignancy. Neuroblastoma-associated transcript 1 (NBAT1) activity is downregulated in NSCLC cells, which inhibits autophagy. Moreover, the association of NBAT1 with proteasome 26S subtype, non-ATPase 10 (PSMD10) improves autophagic disintegration (56). The invasion, growth, along autophagy of bladder malignancy cells are all favorably connected with enhanced activity of the small nucleolar RNA host gene 1 (SNHG1), which acts via the ATG14/miR-493-5p trail (57). HOXA transcript antisense RNA, myeloid-specific 1 (HOTAIRM1) enhances growth as well as autophagy of AML cells. Moreover, nuclear HOTAIRM1 facilitates early growth response 1 (EGR1) breakdown by acting as a support for promoting MDM2 (Murine double minute 2)-EGR1 complex development. On the other hand, cytoplasmic HOTAIRM1 serves as a decoy for miR-152-3p, thereby enhancing the activity of ULK3 (Unc-51 resembling kinase 3) (58). Opa-interacting protein 5 antisense RNA 1 (OIP5-AS1) overexpression improves autophagy in CML K562 cells, but its suppression reduces autophagy and increases imatinib susceptibility. Furthermore, it improves autophagy-associated imatinib resilience in CML cells via miR-30e-5p/ATG12 alliance (59). Metformin enhances SNHG7 expression, facilitates autophagy in ovarian malignancy cells, and reduces the survival of ovarian malignancy cells treated with paclitaxel. Paclitaxel is more likely to affect these cells because metformin increases the miR-3127-5p/SNHG7 alliance, which controls autophagy (60). Autophagy in PCa cells is extensively inhibited when PCa docetaxel resistance-associated lncRNA1 (PCDRlnc1) levels are reduced. PCDRlnc1 networks with ubiquitin-resembling plant homeodomain as well as ring finger domain 1 (UHRF1), thereby stimulating its expression in PCa cells, which activates autophagy-associated Beclin-1 protein (61). Downregulating lncRNA RP11-476D10.1 levels increases autophagy along with apoptosis in papillary thyroid malignancy cells. This lncRNA interrelates with miR-138-5p, thereby facilitating the activity of leucine-rich repeat kinase 2 (LRRK2) (62). The upregulation of LINC01207 levels in OSCC cells facilitates apoptosis along with autophagy. Furthermore, lactate dehydrogenase A (LDHA)/miR-1301-3p/LINC01207 governing alliance stimulates OSCC cell growth (63). SNHG15 focuses on GDNF family receptor alpha 1 (GFRA1)/miR-381-3p activity to increase autophagy, thereby improving the confrontation of osteosarcoma cells to doxorubicin. SNHG15 is increased in doxorubicin-resilient cell lines, and the elimination of SNHG15 activity prevents autophagy as well as the growth of osteosarcoma cells (64). ClRN1 antisense RNA 1 (CLRN1-AS1) suppresses prolactinoma autophagy and growth while forkhead box protein P1 (FOXP1) activates CLRN1-AS1 activity that retains miR-217 as well as influences pituitary prolactinoma cell activity via β-catenin signaling trail/Wnt/Dickkopf WNT signaling trail inhibitor 1 (DKK1) (65). Gemcitabine-resilient pancreatic malignancy cell lines have increased PVT1 oncogene expression, which enhances ATG14 activity and Pygopus genus PHD finger 2 (Pygo2). The β-catenin/Wnt/miR-619-5p/PVT1 alliance stimulates cellular autophagy, thereby reducing gemcitabine confrontation (66).

## lncRNAs affecting melanoma drug resistance and therapeutic reaction

Various studies have demonstrated that lncRNAs may affect melanoma cell responses to various treatment modalities including immunotherapy, targeted therapies, and chemotherapy, thereby regulating the development of medication confrontation. Silencing of the lncRNA H19 renders cisplatin-resilient melanoma cell (MC) lines more susceptible to this medication. H19 suppression prevents cisplatin-resilient MCs from forming colonies and induces apoptosis, which is reversed by IGF1 overexpression or miR-18b suppression (67).

MCs susceptible to dacarbazine (DTIC) have increased expression of LINC01158. MCs that overexpress LINC01158 can withstand DTIC medication, but depletion of LINC01158 reinstates susceptibility to this medication (68). Suppression of TUG1, which is overexpressed in melanoma, improves the chemosensitivity of human MCs cell line to 5-fluorouracil (5-FU), and DDP *in vitro* and slows the growth of tumors *in vivo* (69).

Enhanced expression of XIST serves as a predictive indicator of oxaliplatin therapy failure (70). The platinum-based drug displays reduced poisonousness in comparison to cisplatin and is successful in electrochemotherapy for murine melanoma cases (71). Numerous clinical trials have lately explored its efficacy in treating people with melanoma.

Kolenda et al. carried out a retrospective clinical trial wherein plasma samples from patients receiving vemurafenib for BRAF-mutated melanoma were used to assess the expression of 90 lncRNA that may have an association with the development and growth of cancer (72). Three lncRNAs are potential predictive indicators of the effectiveness of vemurafenib therapy in persons with melanoma: IGF2 antisense (IGF2AS), MEG3, along with zinc finger AE-binding homeobox 2-natural antisense transcript (Zeb2NAT) (72).

It has been determined that RMEL3 is a melanoma-controlled lncRNA whose activity is strongly associated with mutations in BRAF^V600E^ and NRAS^Q61L^ (73, 74). The lncRNA is an advantageous modulator of MAPK as well as PI3K signaling in melanoma because its suppression raises PTEN extents, as well as lowers, activated AKT, ERK, and RAF extents (73, 75). In BRAFV600E melanoma cell lines, RMEL3 depletion considerably decreases colony-forming potential. Furthermore, almost 70% of the investigated sequences of RMEL3 present in TCGA display the UV mutational pattern, which consists of C > T replacements in dipyrimidine positions, like CC > TT. These mutations have been correlated to low patient existence rates, although they have no association with RMEL3 expression (73). In the end, vemurafenib, an inhibitor of BRAF^V600E^, considerably reduces the expression of RMEL3, causing an increase in FOXD3 and a reduction in ERK phosphorylation (73).

MCs that have developed resistance to the BRAF^V600E^ inhibitor PLX4720 have a substantial downregulation of TLSCN8 lncRNA. Furthermore, TSLNC8 reduction prevents apoptosis in melanoma cells sensitive to BRAF inhibitors following PLX4720 therapy (76). MIRAT displays overexpression in numerous NRAS-mutated MC lines resilient to the MEK surpassing agent trametinib. Moreover, its presence shows a dose- and time-reliance on enhancement following trametinib therapy (77).

The activity of MOB kinase stimulator 3B (MOB3B) in MCs is enhanced by the induction of EMICERI (EQTN MOB3B IFNK C9orf72 enhancer RNA I) lncRNA. A paralog of MOB1A/B kinases, MOB3B is a beneficial modulator of the Hippo signaling trail whose stimulation results in vemurafenib resilience (78). Large tumor suppressor kinase 1 (LATS1) is downregulated when MOB3B is overexpressed, which triggers the Hippo signaling trail (79).

Numerous studies demonstrate that different lncRNAs, including NKILA, NEAT1, and XIST, are typically engaged in controlling the human immune system as well as infiltrating tumor-immune cells (80–82). Recently, immune-associated lncRNAs have been identified via the TCGA dataset known as SKCM, which includes clinical and molecular data for 470 melanoma patients (83, 84). Ping et al. identified 28 immune-associated lncRNAs in melanoma-affected persons employing an altered least absolute shrinkage and selection operator (LASSO) regression model. Of these, 17 pairings of co-expressed lncRNAs can separate the SKCM populace into low and high-risk groups (83). Such as, in the SKCM model, co-expression of the lncRNA pair U62631.1 and MIR205HG is related to an increased risk factor (*p-value < 0.001*) while co-expression of the class II primary histocompatibility complex, DQ beta 1 antisense 1 (HLA-DQB1-AS1), and ubiquitin-resembling activating enzyme 6 antisense 1 (UBA6-AS1) is associated with a shielding effect (*p-value < 0.001*). Furthermore, there is a relationship (*p-value* < 0.01) between the high-risk set and the activity of particular mutant genes, like KIT and BRAF (83). Wang et al. identified eight immune-associated lncRNAs with predictive value in the SKCM cohort using the Cox regression model and survival analysis (84). Moreover, the expression of MIR205HG is associated with a poor result (*p-value* < 0.001), while HLA-DQB1-AS1 is associated with immune defense (*p-value* = 0.048). Despite the convergent findings in the studies, these 2 computational techniques possess constraints: it is imperative to carry out more clinical trials involving a large number of melanoma patients. Furthermore, the raw data from the TCGA SKCM is not sufficient to make inferences about novel melanoma biomarkers.

Lastly, in adenocarcinoma and melanoma, the presence of MIR155HG is favorably correlated with the presence of immune checkpoint genes like TIM3, CTLA4, LAG3, and PD-1 (85), and increased circ_0020710 expression. Therefore, cytotoxic lymphocyte depletion as well as the resilience to anti-PD-1 melanoma therapy are associated with CXCL12 increase (86). As a result, immune checkpoint inhibitor-based melanoma immunotherapies may be compromised by increased expression of circ_0020710 and MIR155HG.

In melanoma treatment, several lncRNAs may also be taken as direct targets. Mice treated with intravenous and intratumor doses of SAMMSON antisense nucleotides showed an extensive inhibition of tumor development. Furthermore, mice exposed to dabrafenib and SAMMSON antisense nucleotides have demonstrated tumor shrinkage, with no significant adverse effects or weight loss observed, unlike mice subjected to a regimen comprising dabrafenib and trametinib (87). The findings demonstrate that SAMMSON can be a significant selective therapeutic site against melanoma and a diagnostic tool for cancer. Furthermore, the lowering of SAMMSON considerably lowers the clonogenicity of the malignancy cells that express it, irrespective of their BRAF, NRAS, or TP53 status. In drug-resistant cell lines, this improves the cytotoxic impact of vemurafenib along with the MEK inhibitor pimasertib (87). Furthermore, one of the mechanisms that give melanoma cells resistance to directed therapy in contradiction of the MAPK trail may be the transcriptional activation of SAMMSON by SOX10 through vemurafenib. SAMMSON deletion caused p53 signaling and made BRAF-mutant melanoma more susceptible to BRAF inhibitors both *in vivo* and *in vitro* (88).

Particular antisense oligonucleotides (ASO) are employed in several solid tumors like melanoma, ovarian, prostate, breast, and lung malignancies for targeting mitochondria-resultant nuclear lncRNAs, including antisense non-coding mitochondrial RNAs ASncmtRNA-1 as well as ASncmtRNA-2 (89). *In vivo* and *in vitro* models of murine melanoma, suppression of both ASncmtRNAs by ASO results in suppression of cell growth, thereby promoting apoptosis (90, 91). An ASO medication, Andes-1537, has been evaluated in 2 clinical studies targeting progressive solid tumors, including cervical, pancreatic, and gastric malignancies: NCT02508441 (first submitted on July 15, 2015. It met quality control criteria on July 23, 2015, and was first presented on July 27, 2015​ (ICHGCP)​​ (ANDES | BIOTECHNOLOGIES)​) as well as NCT03985072 (employing).

Resveratrol, quercetin, genistein, and curcumin are examples of phytochemicals that have been shown to have anti-tumor impacts. This is another method of treating lncRNAs. These and other substances may be considered potential anti-cancer medications since it has been revealed that they influence the expression of lncRNAs linked to cancer (92).

## Long non-coding RNA as a possible biomarker for prognosis and diagnosis in melanoma

Several reports have demonstrated that lncRNAs are important for many biological processes in various malignancies, like melanoma. Based on research, lncRNAs are useful as a biomarker for melanoma prompt identification, prognosis, as well as therapy (19, 93–95). Recently, a variety of RNA variants have been used as biomarkers to identify disorders (96, 97). However, there are still very few specialized detection panels. Finding common lncRNA dysregulation may provide new therapy targets and provide insight into the detection and prognosis of the early patient. Many studies have demonstrated over- or under-expression of particular types of lncRNAs, such as HOXA6, FDG5-AS1, PVT1, and NKILA in patients with melanoma as a result of developments in sequencing methods (72, 98, 99). However, demonstrating its detection efficacy is crucial for the clinical applicability of biomarkers. Furthermore, for upcoming clinical applications, these biomarkers’ predictive accuracy needs to be determined (72).

**Figure 4:**
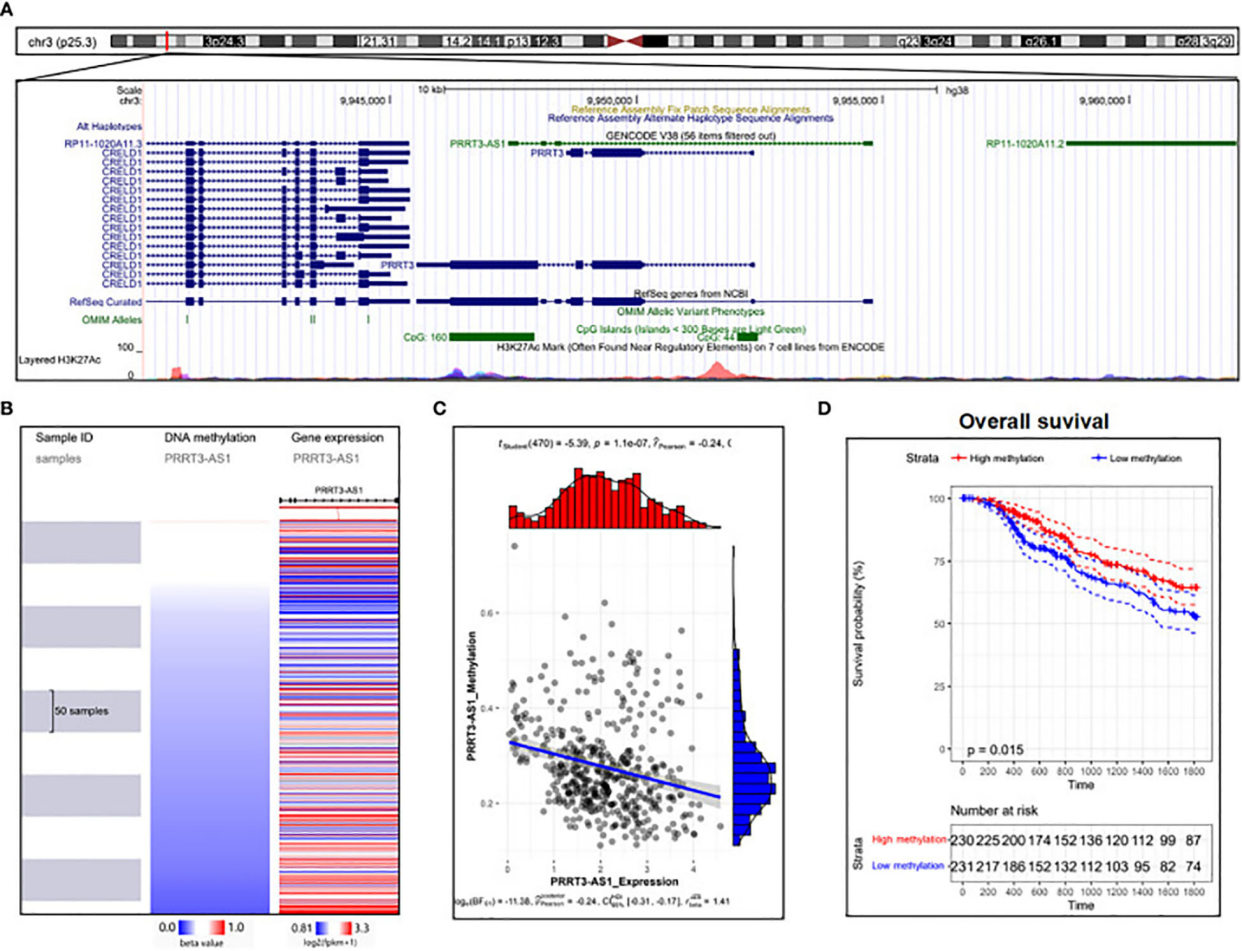
DNA methylation analysis of PRRT3-AS1 in TCGA-SKCM. **(A)** PRRT3-AS1 CpG islands in the human genome. **(B)** PRRT3-AS1 expression and methylation distribution. **(C)** The expression level of PRRT3-AS1 and the distribution of methylation. **(D)** Survival analysis using the Kaplan–Meier curve between the high-methylation and low-methylation groups. Reproduced with permission from (103).

The role of lncRNAs in melanoma has been investigated in numerous studies using melanoma cell lines. In a study by Bian et al., NKILA decreased in melanoma tissue, thereby, reducing the development of the cell cycle and growth. Moreover, NKILA effectively reduced invasion and triggered apoptosis in melanoma cell lines by controlling the nuclear factor kappa B (NF-θB) signaling trail (98). The decreased expression of HOTAIR was found to be linked to melanoma cell growth inhibition and apoptosis induction via NF-/B control (100). HOTAIR increases the development and spread of melanoma cells by absorbing miR-152-3p, thereby triggering the PI3k/Akt/mTOR signaling cascade (101). Investigating the lncRNA PVT1 mechanism in uveal melanoma (UM) cell lines, it was found that suppressing lncRNA PVT1 effectively reduced the clonogenic ability of the cells. Furthermore, it was shown that EZH2 expression was downregulated by PVT1 suppression, which suppressed UM cell growth and enhanced apoptosis (102). PRRT3-AS1 has been proposed as a possible diagnostic and predictive indicator for melanoma by Zhang et al., employing data from the GEO, and TCGA databases (**Figure 4**) (103).

## LncRNAs as melanoma biomarkers

Cells can release LncRNAs, which can be identified in a variety of bodily fluids including urine, serum/plasma, and blood (104). They are either released by living cells via extracellular vesicles, or they come from apoptotic and necrotic cells. Since secreted vesicles shield LncRNAs from the destruction of RNAses. As a result, they become excellent contenders for persistent prognostic or detection markers (105).

Patients with melanoma have been found to have multiple circulating LncRNAs. Patients with advanced melanoma have plasma lncRNA HOTAIR, and there is a significant association between the state of the tumor and HOTAIR expression in melanoma tumors (106). The lncRNA LINC01638 is considerably overexpressed in the plasma of malignancy patients, indicating a normal recurrence (107). The SPRY4-IT1 activity is enhanced in the plasma of melanoma-diseased persons in comparison to healthy persons, with its levels showing a strong correlation with tumor spot along with state (108). Furthermore, the plasmacytoma variant translocation 1 (PVT1) lncRNA was found as enhanced in the serum of melanoma-affected individuals, with its activity corresponding with tumor state as well as serving as an indicator of postoperative disorder dynamics (109). Kolenda et al. found a signature of 17 lncRNAs in the plasma of melanoma-affected persons, which serves to differentiate between healthy persons and those with melanoma. These three LncRNAs- ZEB2-AS1, MEG3, IGF2AS—were recognized as separate predictive elements in *BRAF*-mutant progressive melanoma-affected persons sera cured using vemurafenib (72).

## Combination of circulating lncRNAs for improved diagnostic efficacy and emerging tools for enhanced lncRNA identification

Numerous reports have integrated the identification potential of multiple circulating lncRNAs to improve their diagnostic performance while compensating for the poor sensitivity/specificity of some lncRNAs. Hu et al., incorporated lncRNAs SPRY4-IT1, NEAT1, and ANRIL on nonsmall-cell lung (nscl) cancer, achieving an AUC (ROC) (area under the ROC curve - receiver operating characteristic), sensitivity, and selectivity of 0.876, 82.8%, and 92.3% respectively (110). Serum XIST and HIF1A-AS1 collectively also showed good nonsmall-cell lung cancer detection capabilities (111). SPRY4-IT1 demonstrated a specificity and sensitivity of 89,4% and 72.8% respectively (AUC: 0.842) in the identification of esophageal squamous cell carcinoma (ESCC) when paired with POU3F3 and HNF1AAS1 (112). Yu et al. demonstrated the combining circulating lncRNAs PVT1 along with uc002mbe.2 indicated the existence of hepatocellular malignancy featuring a sensitivity and selectivity of 60.5% and 90.6% respectively (113). The combined assessment of plasma concentrations of LOC152578, XLOC_006844, as well as XLOC_000303 facilitated the identification of colorectal malignancy, achieving AUC (0.975), a sensitivity (80%), and a specificity (84%) (114). Further instances such as the integration of lncRNAs RP11-160H22.5, LOC149086, and XLOC_014172 achieved a specificity and sensitivity of 73% and 82% respectively (AUC: 0.896) for the identification of hepatocellular carcinoma (115). Several reports have explored the identification pattern of higher in comparison to 3 circulating lncRNAs. In another study, Yan et al., demonstrated that a 4-lncRNA ensemble, inclusive of PEG10, ESCCAL-1, POU3F3, and UCA1, presents an outstanding diagnostic approach for the precise identification of ESCC. This multi-lncRNA composite effectively discriminates ESCC patients from healthy individuals, achieving AUC, specificity, and a sensitivity of 0.853, 80.20%, and 80.20% respectively (116). They showed that the 4-lncRNA group outperformed each lncRNA in terms of diagnostic efficacy, hence confirming the clinical importance of using such a combination method. Furthermore, Zhang et al. found a set of 5 plasma lncRNAs (TINCR, BANCR, LINC00857, CCAT2, and AOC4P) that outperformed CEA biomarkers for distinguishing GC-affected persons from healthy individuals achieving an AUC of 0.91 (117). Wu et al. demonstrated that a pattern of five lncRNAs displays the capacity to effectively discriminate serum specimens from patients afflicted with renal cell carcinoma (RCC) when contrasted with samples obtained from healthy persons (118). The amalgamation of lncRNA-LET, PANDAR, PVT1, PTENP1, as well as linc00963, discriminated RCC specimens having an AUC of 0.823. Particularly, none of the individual lncRNAs displayed the same level of diagnostic accuracy as the composite 5-lncRNA pattern. Plasma specimens from patients having pancreatic ductal adenocarcinoma have been investigated for PANDAR and PVT1 within an 8-lncRNA signature (119). A customized nCounter Expression Assay (Nanostring Technologies, USA) that enables simultaneous qPCR analysis employing TaqMan probes was employed to identify the 8-lncRNA pattern. Enhanced diagnostic efficacy can potentially be achieved by the enhanced identification of lncRNAs in human specimens. Recently, new and significantly sensitive techniques have emerged to address this objective. Chen et al. established a new biocompatible electrochemical biosensor called “SPCE Au NCs/MWCNT-NH2” for the identification of lncRNA MALAT1 in nscl cancer (120). They emphasized that this innovative approach has numerous significant advantages over conventional RT-PCR, such as rapid recognition, cheaper costs, and ease of use. Morlion et al. established a novel custom lncRNA sequencing method employing an ensemble of 565,878 targeting agents targeting 49,372 human lncRNA genes. This approach showed enhanced detection sensitivity (121). The use of a custom enrichment strategy represents a major advancement in the field of lncRNA identification meanwhile it facilitates the identification of a large number of lncRNAs with greater repeatability and accuracy in comparison to traditional total RNA-sequencing techniques.

In summary, the composite signature formed by integrating multiple blood-based lncRNAs is purportedly superior in diagnostic efficiency compared to distinct circulating lncRNAs. Concurrently, the advent of novel approaches heralds promising prospects for enhanced recognition of lncRNAs in human biofluids.

## Conclusion and future prospects

Research efforts in clinical and fundamental oncology, addressing cancer as a significant global public health concern, have yielded numerous advancements in recent times. However, the challenge of effectively managing tumor-related morbidity and mortality persists. The primary and paramount objective of tumor research is to develop strategies that precisely target and eliminate tumor cells while safeguarding usual cells from harm. As our comprehension of intracellular molecules advances, numerous molecules involved in tumor progression have been identified. Particularly, non-coding RNAs are significant in cell death, significantly influencing tumor onset and growth. However, controlled cell death may have rather distinct biological functions in various biological contexts. The majority of the genes associated with cellular death in cancer have not had their functions well investigated and the signal of controlled cell demise in tumors also remains unclear. Thus, understanding the modulating trials of ncRNAs in cancer-linked cellular demise, detecting significant therapeutic targets for cell death in cancer, and creating new immunotherapies relying on these non-coding RNAs are of immense and lasting importance in the fight against cancer.

The biology of lncRNAs with a specific focus on recent findings pertinent to managing melanoma was explored. LncRNA investigation is progressing rapidly. At present, 13 lncRNA genes are found in the pathogenesis of melanoma. Their particular expression designs in certain cell or tumor types enable them promising contenders for diagnostic markers or therapeutic targets.

Markers that identify or track the response to novel and costly treatments for metastatic melanoma are also needed, in addition to innovative, more potent, and less toxic therapeutic approaches. Further study is required to explore the role of lncRNAs in achieving this goal.

One drawback of the currently available data is that inconsistent findings have been documented for various lncRNAs. For instance, Tian et al. (122) found no statistically major variance in HOTAIR expression between major melanoma specimens and nearby usual tissues, although the data-mining of publicly accessible gene expression data displayed an enhanced level of HOTAIR presence in melanomas in comparison to nontumor tissues (123). The framework of the study and the experimental procedures may have further restrictions. Frequently, there are not enough samples to reach definitive inferences, or the cell lines that were chosen are only typical of a small portion of the melanomas that individuals have been reported to have. Findings derived from underpowered experiments require sovereign validation through adequately powered experiments before advancing scientific understanding.

In conclusion, melanoma serves as a prime example of the promptly advancing understanding of the significance of lncRNAs in oncology. One or more lncRNAs may be adopted for use as melanoma biomarkers in the near future, provided there is sufficient clinical validation. This approach has already been put into practice in prostate malignancy through the incorporation of lncRNA PCA3 into a commercially available urine-based diagnostic assay (124). Further applications could be the therapeutic implications of this study. Similar to miRNAs, inhibiting lncRNAs might also hold therapeutic promise. The considerable tumor selectivity of a number of the lncRNAs may be a major factor in the success of this effort.

In summary, although the investigation of circulating lncRNAs is in its nascent phase, the global surge in interest surrounding lncRNAs along with the advent of novel tools to enhance their identification, specificity, and clinical applicability undoubtedly enhance the likelihood of eventually identifying dependable blood-based biomarkers. These biomarkers hold the potential for early and precise cancer identification across various types of malignancies.
